# An efficient method for generalized linear multiplicative programming problem with multiplicative constraints

**DOI:** 10.1186/s40064-016-2984-9

**Published:** 2016-08-09

**Authors:** Yingfeng Zhao, Sanyang Liu

**Affiliations:** 1 School of Mathematics and Statistics, Xidian University, Xi’an, 710071 China; 2 School of Mathematical Science, Henan Institute of Science and Technology, Xinxiang, 453003 China

**Keywords:** Generalized linear multiplicative programming, Global optimization, Branch and bound, 90C26, 90C30

## Abstract

We present a practical branch and bound algorithm for globally solving generalized linear multiplicative programming problem with multiplicative constraints. To solve the problem, a relaxation programming problem which is equivalent to a linear programming is proposed by utilizing a new two-phase relaxation technique. In the algorithm, lower and upper bounds are simultaneously obtained by solving some linear relaxation programming problems. Global convergence has been proved and results of some sample examples and a small random experiment show that the proposed algorithm is feasible and efficient.

## Background

Multiplicative programming refers to a class of optimization problems which contains products of real functions in objective and (or) constraint functions. In this study, we consider the following generalized linear multiplicative programming problem:

$$\begin{aligned} {\mathrm{(GLMP)}:}\left\{ \begin{array}{ll}{\mathrm{min}} &{} f_0(x)=\sum \nolimits _{j=1}^{p_0}f_{0j}(x)\\ {\mathrm{s.t.}} &{} f_i(x)=\sum \nolimits _{j=1}^{p_i}f_{ij}(x)\le 0,\quad i=1,2,\dots ,M,\\ &{} f_i(x)=\sum \nolimits _{j=1}^{p_i}f_{ij}(x)\ge 0,\quad i=M+1,M+2,\dots ,N,\\ &{} x\in D=\{x\in R^n \mid Ax \le b,\,\,x\ge 0\}, \end{array} \right. \end{aligned}$$

where $$f_{ij}(x)=\phi _{ij}(x)\psi _{ij}(x), \phi _{ij}(x)=\sum \nolimits _{k=1}^{n} a_{ijk}x_k +b_{ij},$$$$\psi _{ij}(x)=\sum \nolimits _{k=1}^{n} c_{ijk}x_k +d_{ij},$$ while the coefficients $$a_{ijk},$$$$c_{ijk}$$ and the constant terms $$b_{ij},$$$$d_{ij}$$ are all arbitrary real numbers, $$i=0,1,2,\ldots ,N,$$$$j=1,2,\ldots ,p_i,$$$$k=1,2,\ldots ,n$$; $$A \in R^{m \times n}$$ is a matrix, $$b \in R^m$$ is a vector, set *D* is nonempty and bounded.

Generalized linear multiplicative programming (GLMP) with multiplicative objective and constraint functions is a special case of multiplicative programming. It has attracted considerable attention of researchers and practitioners for many years. This is mainly because, from the practical point of view, it possesses important application foreground in various fields, including microeconomics (Henderson and Quandt 1961), multiple-objective decision (Benson 1979; Keeney and Raiffa 1993; Geoffrion 1967), plant layout design (Quesada and Grossmann 1996), data mining$\backslash$pattern recognition (Bennett and Mangasarian 1994), marketing and service planning (Samadi et al. 2013), robust optimization (Mulvey et al. 1995), and so on. And from the algorithmic design point of view, a product of two affine functions, as noted in Avriel et al. (2010), need not be convex(even not be quasi-convex), and hence problem (GLMP) is a global optimization problem which may have multiple non-global local solutions, global solution methods for problem (GLMP) have great difficulties and challenges. Due to the facts above, design efficient solution methods for globally solving the (GLMP) has important theoretical and the practical significance.

In the past few decades, many solution methods have been devised for solving the problem (GLMP). These methods are mainly classified as parameter-based methods (Konno et al. 1994; Thoai 1991), outer-approximation methods (Gao et al. 2006; Kuno et al. 1993), outcome-space cutting plane methods (Benson and Boger 2000), branch-and-bound methods (Ryoo and Sahinidis 2003; Shen and Jiao 2006; Konno and Fukaishi 2000; Kuno 2001) and various heuristic methods (Benson and Boger 1997; Liu et al. 1999; Fan et al. 2016). Recently, Wang proposes a global optimization algorithm for a kind of generalized linear multiplicative programming by using simplicial partition techniques (Wang et al. 2012), but his method is only valid for problems in which the constraint functions are all linear. Jiao and Liu (2015) present an effective algorithm for solving the generalized linear multiplicative problem with generalized polynomial constraints by converting it into an equivalent generalized geometric programming problem, the problem they considered is more general but only valid under the assumption $\phi _{ij}(x)>0, \psi _{ij}(x)>0, \forall x \in X$. There are many other solution methods not mentioned for (GLMP) and its special case, nevertheless, most of these methods are either developed for special circumstances or can only obtain a local solution of problem (GLMP).

In this paper, we put forward a fast global optimization algorithm for generalized linear multiplicative programming problem (GLMP). Our research can be divided into three steps. First, a well performed linear relaxation programming problem for the (GLMP) is established by using a new two-phase relaxation technique. Second, two key operations for developing a branch and bound algorithm for the (GLMP) are described. Finally, global convergence property is proved and some numerical experiments are executed to illustrate the feasibility and robustness of the proposed algorithm. Compared with some existing methods, the new two-phase relaxation technique we used in the algorithm has a very good approximation effect, and it doesn’t require the condition $\phi _{ij}(x)>0, \psi _{ij}(x)>0, \forall x \in X$. Further more, relative to the algorithm in Jiao (2009), Quesada and Grossmann (1996), the proposed algorithm can be applied to a more general case of linear multiplicative programming problem.

The reminder of this article is arranged as follows. Section "Two-phase relaxation technique" explains how the two-phase relaxation method is realized, section "Algorithm and its convergence" introduces the branch and bound operation for deriving the presented algorithm. The algorithm statement as well as the convergence property are described in section "Numerical experiments". In section "Concluding remarks", the results of some numerical experiments appeared in recent literatures are listed and some concluding remarks are reported in the last section.

## Two-phase relaxation technique

As is known to all, construct a well performed relaxation problem can bing great convenience for designing branch and bound algorithm of global optimization problems. In this section, equivalent transformation technique and a new two-phase relaxation skill will be used to establish a linear relaxation programming problem for underestimating the optimal value of problem (GLMP).

First, we compute the initial variable bounds by solving the following linear programming problems:

$$\begin{aligned} x_{i}^{l}=\min \limits _{x\in D}x_i, \quad x_{i}^{u}=\max \limits _{x\in D}x_i, \quad i=1,2,\ldots ,n, \end{aligned}$$

then an initial rectangle $X^0=\left\{ x \in R^n \mid x_{i}^{l} \le x_{i} \le x_{i}^{u},\quad i=1,2,\ldots ,n\right\}$ will be obtained. To construct the first-phase relaxation programming problem of the (GLMP) over sub-rectangle $X \subset X^{0}$, we further solve some linear programming problems as follows:

1 $$\begin{aligned} \begin{aligned} l_{ij}=\min \limits _{x\in D \bigcap X}\phi _{ij}(x),&\quad u_{ij}=\max \limits _{x\in D \bigcap X}\phi _{ij}(x), \\ L_{ij}=\min \limits _{x\in D \bigcap X}\psi _{ij}(x),&\quad U_{ij}=\max \limits _{x\in D \bigcap X}\psi _{ij}(x). \end{aligned} \end{aligned}$$

Upon criteria (1), it is clear that

2 $$\begin{aligned} \left( \phi _{ij}(x)-l_{ij}\right) \left( \psi _{ij}(x)-L_{ij}\right) \ge 0,\quad \left( \phi _{ij}(x)-u_{ij}\right) \left( \psi _{ij}(x)-U_{ij}\right) \ge 0 , \end{aligned}$$

and

3 $$\begin{aligned} \left( \phi _{ij}(x)-l_{ij}\right) \left( \psi _{ij}(x)-U_{ij}\right) \le 0,\quad \left( \phi _{ij}(x)-u_{ij}\right) \left( \psi _{ij}(x)-L_{ij}\right) \le 0 , \end{aligned}$$

by taking (2) and (3) together, we have

4 $$\begin{aligned} \phi _{ij}(x) \psi _{ij}(x) \ge \max \left\{ u_{ij}\psi _{ij}(x)+U_{ij}\phi _{ij}(x)-U_{ij}u_{ij},\,l_{ij}\psi _{ij}(x)+L_{ij}\phi _{ij}(x)-L_{ij}l_{ij}\right\} , \end{aligned}$$

and

5 $$\begin{aligned} \phi _{ij}(x) \psi _{ij}(x) \le \min \left\{ u_{ij}\psi _{ij}(x)+L_{ij}\phi _{ij}(x)-u_{ij}L_{ij},\,l_{ij}\psi _{ij}(x)+U_{ij}\phi _{ij}(x)-U_{ij}l_{ij}\right\} . \end{aligned}$$

For each $i=0,1,2,\ldots ,N,$ by denoting

$$\begin{aligned}\underline{g}^1_{ij}(x) \triangleq u_{ij}\psi _{ij}(x)+U_{ij}\phi _{ij}(x)-U_{ij}u_{ij}, \quad \underline{g}^2_{ij}(x)\triangleq l_{ij}\psi _{ij}(x)+L_{ij}\phi _{ij}(x)-L_{ij}l_{ij},\end{aligned}$$

and

$$\begin{aligned} \overline{g}^1_{ij}(x)\triangleq u_{ij}\psi _{ij}(x)+L_{ij}\phi _{ij}(x)-u_{ij}L_{ij},\quad \overline{g}^2_{ij}(x)\triangleq l_{ij}\psi _{ij}(x)+U_{ij}\phi _{ij}(x)-U_{ij}l_{ij}, \end{aligned}$$

conclusion (4) and (5) can be expressed as

6 $$\begin{aligned} \phi _{ij}(x) \psi _{ij}(x) \le \min \left\{ \overline{g}_{ij}^1(x), \overline{g}_{ij}^2(x) \right\} \triangleq \overline{g}_{ij}(x), \end{aligned}$$

and

7 $$\begin{aligned} \phi _{ij}(x) \psi _{ij}(x) \ge \max \left\{ \underline{g}_{ij}^1(x), \underline{g}_{ij}^2(x) \right\} \triangleq \underline{g}_{ij}(x), \end{aligned}$$

respectively. Then we can obtain a lower bound function $$\underline{g}_i(x)$$ and upper bound function $$\overline{g}_i(x)$$ for $$f_{i}(x)$$, which satisfy $$\underline{g}_i(x) \le f_{i}(x) \le \overline{g}_i(x),$$$$i=0,1,2,\ldots ,N,$$ where

8 $$\begin{aligned} \underline{g}_i(x)=\sum \limits _{j=1}^{p_i}\underline{g}_{ij}(x), \quad \overline{g}_i(x)=\sum \limits _{j=1}^{p_i}\overline{g}_{ij}(x), \quad i=0,1,2,\ldots ,N. \end{aligned}$$

So far, based on the above discussion, we can get the first-phase relaxation programming problem for the (GLMP) which we formulated as follows:

$$\begin{aligned} {\mathrm{(RMP0)}:}\left\{ \begin{array}{ll}{\mathrm{min}} &{} \underline{g}_0(x)=\sum \nolimits _{j=1}^{p_0}\underline{g}_{0j}(x)\\ {\mathrm{s.t.}} &{} \underline{g}_i(x)=\sum \nolimits _{j=1}^{p_i}\underline{g}_{ij}(x)\le 0,\quad i=1,2,\dots ,M,\\ &{} \overline{g}_i(x)=\sum \nolimits _{j=1}^{p_i}\overline{g}_{ij}(x)\ge 0,\quad i=M+1,M+2,\dots ,N,\\ &{} x\in D\bigcap X=\{x\in X \mid Ax \le b,\,\, x\ge 0\}, \end{array} \right. \end{aligned}$$

To get the second-phase linear relaxation programming problem, we will once again relax each nonlinear function appeared in problem (RMP0) according the following conclusion:

9 $$\begin{aligned} \begin{aligned} \underline{g}_i(x)&=\sum \limits _{j=1}^{p_i}\max \left\{ \underline{g}^1_{ij}(x),\,\underline{g}^2_{ij}(x)\right\} \\&\ge \max \left\{ \sum \limits _{j=1}^{p_i}\underline{g}^1_{ij}(x),\,\sum \limits _{j=1}^{p_i}\underline{g}^2_{ij}(x)\right\} \triangleq g_i(x),\,\quad i=0,1,2,\ldots ,M, \end{aligned} \end{aligned}$$

and

10 $$\begin{aligned} \begin{aligned} \overline{g}_i(x)&=\sum \limits _{j=1}^{p_i}\min \left\{ \underline{g}^1_{ij}(x),\,\overline{g}^2_{ij}(x)\right\} \\&\le \min \left\{ \sum \limits _{j=1}^{p_i}\overline{g}^1_{ij}(x),\,\sum \limits _{j=1}^{p_i}\overline{g}^2_{ij}(x)\right\} \triangleq g_i(x),\,\quad i=M+1,M+2,\ldots ,N. \end{aligned} \end{aligned}$$

With conclusion (9) and (10), the second-phase relaxation programming problem (RMP1) of the (GLMP) can be expressed as follows:

$$\begin{aligned} {\mathrm{(RMP1):}}\left\{ \begin{array}{ll} {\mathrm{min}} &{} g_{0}(x)=\max \left\{ \sum \nolimits _{j=1}^{p_0}\underline{g}^1_{0j}(x),\,\sum \nolimits _{j=1}^{p_0}\underline{g}^2_{0j}(x)\right\} \\ {\mathrm{s.t.}} &{} g_i(x)=\max \left\{ \sum \nolimits _{j=1}^{p_i}\underline{g}^1_{ij}(x),\, \sum \nolimits _{j=1}^{p_i}\underline{g}^2_{ij}(x)\right\} \le 0,\quad i=1,2,\dots ,M,\\ &{} g_i(x)=\min \left\{ \sum \nolimits _{j=1}^{p_i}\overline{g}^1_{ij}(x),\, \sum \nolimits _{j=1}^{p_i}\overline{g}^2_{ij}(x)\right\} \ge 0,\quad i=M+1,M+2,\ldots ,N,\\ &{} x\in D \bigcap X=\{x\in X \mid Ax \le b, \quad x\ge 0\}, \end{array} \right. \end{aligned}$$

which is proved equivalent to the following linear programming problem:

$$\begin{aligned} {\mathrm{(ERMP)}:}\left\{ \begin{array}{ll}{\mathrm{min}} &{} t\\ {\mathrm{s.t.}} &{} \sum \nolimits _{j=1}^{p_0}\underline{g}^1_{0j}(x)- t\le 0,\\ &{} \sum \nolimits _{j=1}^{p_0}\underline{g}^2_{0j}(x)- t\le 0,\\ &{} \sum \nolimits _{j=1}^{p_i}\underline{g}^1_{ij}(x)\le 0,\quad i=1,2,\dots ,M,\\ &{} \sum \nolimits _{j=1}^{p_i}\underline{g}^2_{ij}(x)\le 0,\quad i=1,2,\dots ,M,\\ &{} \sum \nolimits _{j=1}^{p_i}\overline{g}^1_{ij}(x)\ge 0,\quad i=M+1,M+2,\dots ,N,\\ &{} \sum \nolimits _{j=1}^{p_i}\overline{g}^2_{ij}(x)\ge 0,\quad i=M+1,M+2,\dots ,N,\\ &{} x\in D \bigcap X=\{x\in X \mid Ax \le b, \quad x\ge 0\}. \end{array} \right. \end{aligned}$$

### **Theorem 1**

*If*$(x^{*},t) \in R^{n+1}$*is a global optimal solution for the (ERMP), then*$x^{*} \in R^n$*is a global optimal solution for the (RMP1). Conversely, if*$x^{*} \in R^n$*is a global optimal solution for the (RMP1), then*$(x^{*},t) \in R^{n+1}$*is a global optimal solution for the (ERMP), where*$t=g_0\left( x^{*}\right)$.

### *Proof*

The proof of this theorem can be easily followed according to the definition of problems (RMP1) and (ERMP), therefore, it is omitted here. $\square$

### **Theorem 2**

*(1) For any*$x \in X$, *we have*

$$\begin{aligned} g_{i}(x) \le f_i(x),\quad i=0,1,2,\ldots ,M, \end{aligned}$$

*and*

$$\begin{aligned} g_{i}(x) \ge f_i(x),\quad i=M+1,M+2,\ldots ,N. \end{aligned}$$

*(2)*$\left| g_i(x)-f_{i}(x)\right| \rightarrow 0$, *as*$\left\| U_i-L_i\right\| \rightarrow 0$, $\left\| u_i-l_i\right\| \rightarrow 0,$*where*$U_i=(U_{i1},U_{i2},\ldots ,U_{i p_i}),L_i=(L_{i1},L_{i2},\ldots ,L_{i p_i})$*and*$u_i=(u_{i1},u_{i2},\ldots ,u_{i p_i}),l_i=(l_{i1},l_{i2},\ldots ,l_{i p_i})$.

### *Proof*

(1) can be easily verified from conclusion (4), (5), (9) and (10), thus the detailed proof is omitted here.

For (2), according to the Cauchy–Schwarz inequality, we know that for $i=0,1,\ldots ,M,$

$$\begin{aligned} \begin{aligned}&\left| g_i(x)-f_{i}(x)\right| \\&=\left| \max \left\{ \sum \limits _{j=1}^{p_i}\underline{g}^1_{ij}(x),\,\sum \limits _{j=1}^{p_i}\underline{g}^2_{ij}(x)\right\} -\sum \limits _{j=1}^{p_i}\phi _{ij}(x)\psi _{ij}(x)\right| \\&=\left| \max \left\{ \sum \limits _{j=1}^{p_i}\underline{g}^1_{ij}(x)-\sum \limits _{j=1}^{p_i}\phi _{ij}(x)\psi _{ij}(x), \,\sum \limits _{j=1}^{p_i}\underline{g}^2_{ij}(x)-\sum \limits _{j=1}^{p_i}\phi _{ij}(x)\psi _{ij}(x)\right\} \right| \\&=\left| \max \left\{ \sum \limits _{j=1}^{p_i}\left( l_{ij}\psi _{ij}(x)+L_{ij}\phi _{ij}(x)-l_{ij}L_{ij}-\phi _{ij}(x)\psi _{ij}(x)\right) ,\right. \right. \\&\left. \left. \,\,\,\,\,\,\,\,\,\,\,\,\,\,\,\,\,\,\,\,\,\,\,\,\,\,\,\,\,\,\,\, \sum \limits _{j=1}^{p_i}\left( u_{ij}\psi _{ij}(x)+U_{ij}\phi _{ij}(x)-U_{ij}u_{ij}-\phi _{ij}(x)\psi _{ij}(x)\right) \right\} \right| \\&=\left| \max \left\{ \sum \limits _{j=1}^{p_i}\bigg (\psi _{ij}(x)-L_{ij}\bigg )\bigg (\phi _{ij}(x) -l_{ij}\bigg ),\sum \limits _{j=1}^{p_i}\bigg (\psi _{ij}(x)-U_{ij}\bigg )\bigg (\phi _{ij} -u_{ij}\bigg )\right\} \right| \\&\le \max \left\{ \left\| (U-L) \right\| \left\| (u-l)\right\| \right\} , \end{aligned} \end{aligned}$$

for the case $i=M+1,M+2,\ldots ,N,$ it can be proved with the similar method, so omitted here, and thus the Proof of Theorem 2 is completed. $\square$

### *Remark 1*

From Theorems 1 and 2, we only need to solve problem (ERMP) instead of solving the (RMP1) to obtain the lower and upper bounds of the optimal value in problem (GLMP).

### *Remark 2*

Based on the continuity of linear function, $\left\| U_i-L_i\right\| \rightarrow 0$ and $\left\| u_i-l_i\right\| \rightarrow 0$ will hold when the diameter of *X* approximate to zero, this indicated that we can perform the branching operation in variable space *X* with the convergence property is guaranteed.

### *Remark 3*

Theorem 2 ensures that problem (ERMP) can infinitely approximate the problem (GLMP), as $\left\| X\right\| \rightarrow 0,$ this will guarantee the global convergence of the proposed algorithm.

## Algorithm and its convergence

In this section, we will describe two key operation for designing an efficient branch and bound algorithm for problem (GLMP), that is, branching and bounding. Then the algorithm steps will be summarized with proof process of global convergence property followed.

### Branching and bounding

The branching operation iteratively subdivides the rectangle *X* into subregions according to an exhaustive partition rule, such that any infinite iterative sequence of partition sets shrinks to a singleton. For this, we shall adopt an standard range bisection approach, which is adequate to insure global convergence of the proposed algorithm. Detailed process is described as follows.

For any region $X=[x^l,x^{u}] \subset X^0$, let $r\in {\mathrm{argmin}}\{x_{i}^{u}-x_{i}^{l}\mid i=1,2,\ldots ,n\}$ and $mid=(x_r^l+x_r^u)\slash 2,$ then the current region *X* can be divided into the following two sub-regions:

$$\begin{aligned}\bar{X}=\left\{ x\in R^n \mid x_{i}^{l}\le x_i \le x_{i}^{u},i \ne r,x_{r}^{l}\le x_r \le mid\right\} ,\end{aligned}$$

and

$$\begin{aligned}\bar{\bar{X}}=\left\{ x\in R^n \mid x_{i}^{l}\le x_i \le x_{i}^{u},i \ne r,mid\le x_r \le x_{r}^{u}\right\} .\end{aligned}$$

For each partition subset *X* generated by the above branching operation, the bounding operation is mainly concentrate on estimating a lower bound *LB*(*X*) and a upper bound *UB*(*X*) for the optimal value of problem (GLMP). This operation is realized by solving the linear relaxation programming problem (ERMP) over all partition sets in the $k_{th}$ iteration, and the one with the smallest optimal value will provide the lower bound for optimal value of problem (GLMP) over the initial region $X^0$. Moreover, since any feasible solution of the relaxation programming problem will also be feasible to the (GLMP), so we can evaluate the initial objective value and make the one with smallest value as a new upper bound if possible.

### Algorithm and its convergence

Based on the former discussion, the algorithm steps can be summarized as follows:

**Step 0** (*Initialization*) Choose convergence tolerance $\epsilon =1 \times 10^{-8}$, set iteration counter $k:=0$ and the initial partition set as $\Omega _0=X^0$. Solve the initial linear relaxation problem (ERMP) over region $X^0$, if the (ERMP) is not feasible then there is no feasible solution for the initial problem. Otherwise, denote the optimal value and solution as $f_{bar}$ and $x_{opt}^0$, respectively. Then we can obtain the initial upper and lower bound of the optimal value for problem (GLMP), that is, $UB:=f_0(x^0_{opt}),\quad \text {and} \quad LB:=f_{bar}$. And then, if $UB-LB<\epsilon$, the algorithm can stop, and $x_{opt}^0$ is the optimal solution of the (GLMP), otherwise proceed to step 1.

**Step 1** (*Branching*) Partition $X^k$ into two new sub-rectangles according to the partition rule described in section “Branching and bounding”. Deleting $X^k$ and add the new nods into the active nods set $\tilde{X}^k$, still denote the set of new partitioned sets as $\tilde{X}^k$.

**Step 2** (*Bounding*) For each subregion still of interest $X^{k \mu } \subseteq X^0, \mu =1,2$, obtain the optimal solution and value for problem (RMFP) by solving the relaxation linear programming problem over $X^{k \mu }$, if $LB(X^{k,\mu })> UB,$ delete $X^{k\mu }$ from $\tilde{X}^k$. Otherwise, we can update the lower and upper bounds: $LB=\min \{LB(X^{k,\mu })\mid \mu =1,2\}$ and $UB=\min \{UB, f(x^{k,\mu })\mid \mu =1,2\}$.

**Step 3** (*Termination*) If $UB-LB \le \epsilon$, the algorithm can be stopped, *UB* is the global optimal value for (GLMP). Otherwise, set $k:=k+1,$ and select the node with the smallest optimal value as the current active node, and return to Step 1.

#### **Theorem 3**

*The proposed algorithm either terminates within finite iterations with an optimal solution for (GLMP) be found, or generates an infinite sequence of iterations such that along any infinite branches of the branch-and-bound tree, any accumulation point of the sequence*$\{x^k\}$*will be the global optimal solution of the (GLMP).*

#### *Proof*

(1) If the proposed algorithm is finite, assume it stops at the $k{\text{th}}$ iteration, $k\ge 0$. From the termination criteria, we know that

$$\begin{aligned} UB-LB \le \epsilon . \end{aligned}$$

Based on the upper bounding technique described in Step 3, it implies that

$$\begin{aligned} f(x^k)-LB \le \epsilon . \end{aligned}$$

Let $v_{opt}$ be the optimal value of problem (GLMP), then by section “Branching and bounding” and Step 3 above, we known that

$$\begin{aligned} UB=f(x^k)\ge v_{opt}\ge LB. \end{aligned}$$

Hence, taken together, it implies that

$$\begin{aligned} v_{opt}+\epsilon \ge LB+\epsilon \ge f(x^k)\ge v_{opt}, \end{aligned}$$

and thus the proof of part (1) is completed.

(2) If the algorithm doesn’t terminate within finite iterations and generates an infinite feasible solution sequence $\{x^k\}$ for the (GLMP) via solving the ${\mathrm{(RMP1)}}$. According to the structure of the proposed algorithm, we have

11 $$\begin{aligned} LB_k \le \min \limits _{x \in X}f_0(x), \end{aligned}$$

assume that:

12 $$\begin{aligned} X^{k} \in \mathop {\arg \min } \limits _{X \in \Omega _k}LB(X), \quad x^{k}=x(X^{k}) \in X^{k} \subseteqq X^{0}. \end{aligned}$$

Horst (1998) has proved that ${LB_k}$ is non-decrease and bounded above by $\min \nolimits _{x \in X}f_0(x),$ thus the existence of the limit $LB:= \lim \nolimits _{k \rightarrow \infty }LB_k \le \min \nolimits _{x \in X}f_0(x)$ can be guaranteed. Further more, since ${x^{k}}$ is a sequence on a compact set, it must have a convergent subsequence. For any accumulation point $\hat{x}$ of $\{x^k\}$, there exists a subsequence of $\{x^{k}\}$ which, without loss of generality, we might still denote as $\{x^{k}\}$ satisfied $\lim \nolimits _{k \rightarrow \infty }x^k =\hat{x}$. With similar method in Tuy (1991), we can easily follow that the subdivision of partition sets in step 1 is exhaustive on $X^0$, and the selection of elements to be partitioned is bound improving, thus there exists a decreasing subsequence $X^r \subset X^k$ where $X^r \in \Omega _{r}$ with $x^r \in X^r$, $LB_r=LB(X^r)=g_{0}(x^r),\lim \nolimits _{r\rightarrow \infty }x^r={\hat{x}}$. Based on the construction process of the relaxation problem, we know that the linear relaxation functions $g_i(x)(i=0,1,\ldots ,N)$ used in problem (RMP1)(and thus for (ERMP)) are strongly consistent on $X^0$, hence it follows that $\lim \nolimits _{k \rightarrow \infty }LB_k =LB=g_0(\hat{x})$. Since $\hat{x}$ is feasible to (GLMP) and combining with (11) we can deduce that $\hat{x}$ is a global solution for the (GLMP). $\square$

## Numerical experiments

**Fig. 1:**
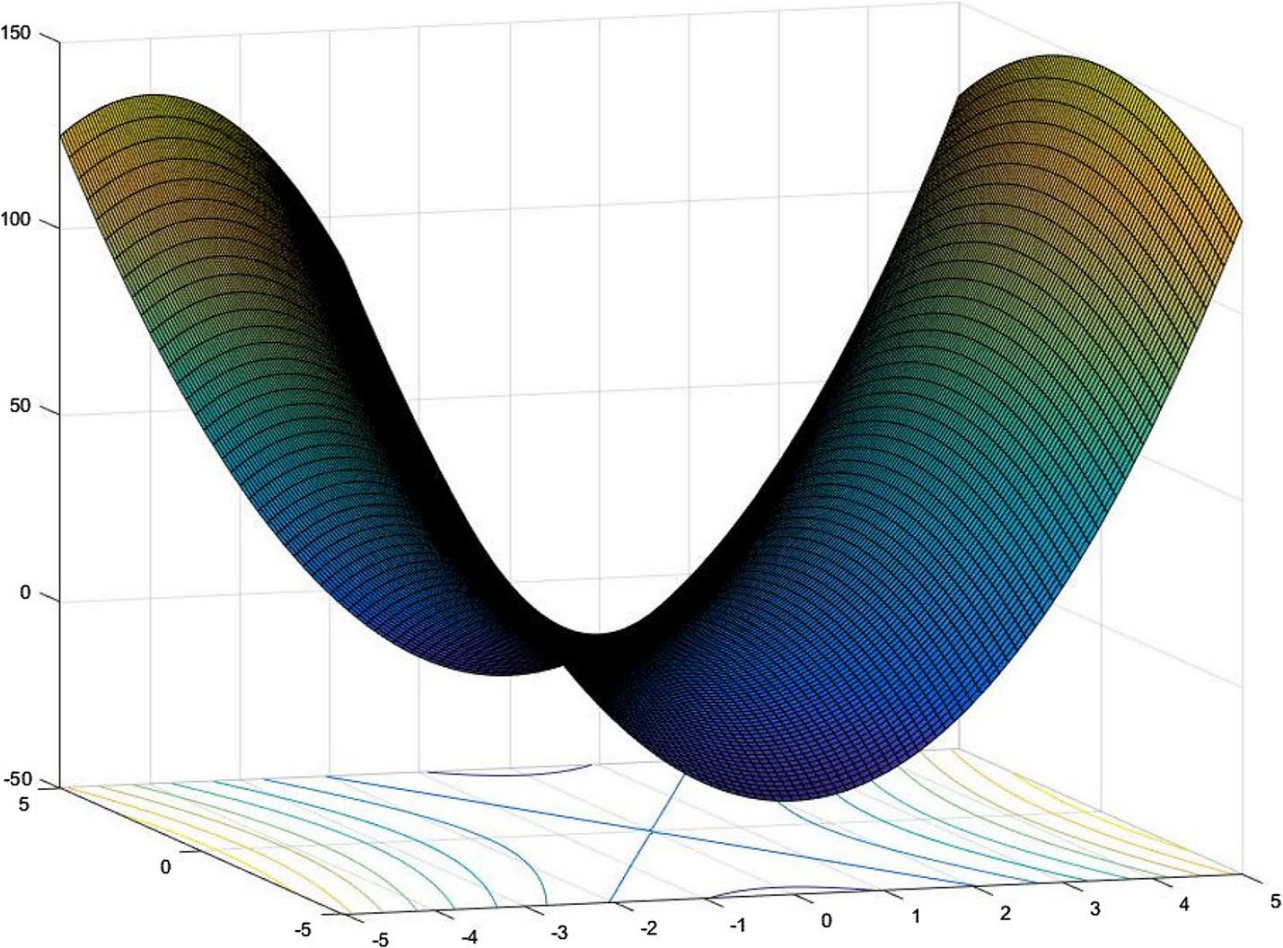
3-D surface and contour plot over [−5, 5; −5, 5] of the objective function in Example 11. From this figure we can see that the objective function in Example 11 may have multiple local optimal solutions over the feasible region

To verify the performance of the proposed algorithm, we solve some test problems in recent literatures (Thoai 1991; Wang et al. 2012; Jiao and Liu 2015; Wang and Liang 2005; Gao et al. 2010; Chen and Jiao 2009; Shen et al. 2008; Shen and Jiao 2006; Jiao 2009) and construct a problem to illustrate the nature that (GLMP) may have multiple local optimal solutions (see Fig. 1), computational results are given in Table 1, where the following notations have been used in row headers: Exa.: the serial number of experiments; Ref.: reference which we contrast with; Opt.Val.: optimal value; Opt.Sol.: optimal solution; Iter: numbers of iterations; Time: CPU time in seconds; Pre.: precision we used in the algorithm. We used the TPRM to represent the two-phase relaxation method given in this paper.

**Table 1:** Results of the numerical contrast experiments 1–11

Exa.	Ref.	Opt. val.	Opt. sol.	Iter	Time	Pre.
1	Wang and Liang (2005)	6.7780	(2.00003, 1.66665)	44	0.18	$10^{-4}$
	Jiao (2009)	6.77778	(2.0, 1.666667)	58	<1	$10^{-8}$
	TPRM	6.77778	(2.0000, 1.6667)	1	0.027	$10^{-8}$
2	Jiao (2009)	−4.0	(2.0, 1.0, 3.0)	43	–	$10^{-8}$
	TPRM	−4.0	(2.0000, 1.0000, 3.0000)	1	0.054	$10^{-8}$
3	Gao et al. (2010)	10.0042	(2.0003, 7.9999)	27	10.83	$10^{-3}$
	Chen and Jiao (2009)	10.00009	(1.999998, 7.9999988)	41	0.02	$10^{-5}$
	TPRM	10.0	(2.0000,8.0000)	2	0.0407	$10^{-8}$
4	Gao et al. (2010)	0.0000	(0.0002, 0.0001, 0, 0, 0, 0, 0, 0, 0, 0, 0)	36	16.03	$10^{-3}$
	TPRM	0.0000	$(0.00, 2.3453, 0.0000, 6.4121, 1.9434, 0.00, 2.4858, 8.4448, 6.9770, 5.8001, 5.1340)$	13	1.2758	$10^{-8}$
5	Thoai (1991)	0.8902	(1.314792, 1.39555, 0, 0.42329)	6	0.1880	$10^{-6}$
	TPRM	0.89019	(1.3148, 0.1396, 0.0000, 0.4233)	1	0.00355	$10^{-8}$
6	Shen et al. (2008)	11.475	(0.61824, 0.0000)	29	0.01	$10^{-3}$
	TPRM	4.0000	(0.0000, 0.0000)	1	0.022	$10^{-8}$
7	Chen and Jiao (2009)	−15.000	(2.0, 1)	1657	120.58	$10^{-6}$
	TPRM	−15.0000	(2.0000, 1.0000)	110	57.224	$10^{-8}$
8	Shen and Jiao (2006)	0.0000	(2.00, 1.00)	24	–	$10^{-3}$
	Jiao and Liu (2015)	0.00000003	(2.0000061, 1.0)	16	0.018	$10^{-8}$
	TPRM	0.0000	(2.0000, 1.0000)	1	0.05406	$10^{-8}$
9	Shen and Jiao (2006)	1.1771	(1.17709, 2.1772)	434	1	$10^{-3}$
	Jiao and Liu (2015)	1.17708	(1.17709, 2.1772)	189	0.226	$10^{-6}$
	TPRM	1.1770	(1.177088, 2.17718)	3	0.66936	$10^{-8}$
10	Jiao and Liu (2015)	3.0000	(0.0000,4.0000)	25	0.750	$10^{-8}$
	TPRM	3.0000	(0.0000,4.0000)	1	0.02456	$10^{-8}$
11	TPRM	−25.0000	(0.0000, −5.0000)	47	22.64563	$10^{-8}$

We coded the algorithms in Matlab 2014a, and ran the tests in a micro computer with Intel(R) Xeon(R) processor of 2.4 GHz, 4 GB of RAM memory, under the Win10 operational system. We used linprog solver to solve all linear programming problems.

Table 1 shows that our algorithm performs more efficient than that in references Ryoo and Sahinidis (2003), Shen and Jiao (2006), Thoai (1991), Tuy (1991), Wang et al. (2012) and Wang and Liang (2005). Especially for Examples 1, 2, 5, 6, 8 and 10, our algorithm only need one iteration to determine the global optimal solutions, this indicates that our new relaxation technique is so efficient that the global optimal solution can be founded in the initialization step. Further more, we constructed an example (Example 11 and Fig. 1) with multiple local optimum to test our algorithm.

### *Example 1*

(Refs. Wang and Liang 2005; Jiao 2009).

$$\begin{aligned} \left\{ \begin{array}{ll} \min &{}x^{2}_1+x^{2}_2\\ {\mathrm {s.t.}} &{} 0.3x_1x_2\ge 1,\\ &{} 2\le x_1\le 5,\\ &{} 1 \le x_2\ge 3, \end{array} \right. \end{aligned}$$

### *Example 2*

(Refs. Jiao 2009).

$$\begin{aligned} \left\{ \begin{array}{ll} \min &{}x^{2}_1+x^{2}_2-x_{3}^{2}\\ {\mathrm {s.t.}} &{} 0.3x_1x_2+0.3x_2x_3+0.6x_1x_3\ge 4,\\ &{} 2 \le x_1 \le 5,\\ &{} 1 \le x_2 \ge 3,\\ &{} 1 \le x_3 \le 3, \end{array} \right. \end{aligned}$$

### *Example 3*

(Refs. Gao et al. 2010; Chen and Jiao 2009).

$$\begin{aligned} \left\{ \begin{array}{ll} \min &{}(x_1+x_2)(x_1-x_2+7)\\ {\mathrm {s.t.}} &{} 2x_1+x_2\le 14,\\ &{} x_1+x_2\le 10,\\ &{} -4x_1+x_2\le 0,\\ &{} 2x_1+x_2\ge 6, \\ &{} x_1+x_2\ge 6,\\ &{} x_1\le 5,\\ &{} x_1+x_2\ge 0,\\ &{} x_1-x_2+7\ge 0. \end{array} \right. \end{aligned}$$

### *Example 4*

(Refs. Gao et al. 2010; Chen and Jiao 2009).

$$\begin{aligned} \left\{ \begin{array}{ll} \min &{}(c_1^Tx+d_1)(c_2^Tx+d_2)\\ {\mathrm {s.t.}} &{} Ax \le b, \end{array} \right. \end{aligned}$$

where

$$\begin{aligned} b&= (81,72, 72, 9, 9, 9, 8, 8)^{T},\quad d_{1}=0,d_{2}=0,\\ c_{1}&= \left(1, 0, \frac{1}{9}, 0, 0, 0, 0 ,0, 0, 0, 0\right)^{T},\quad c_{2}=\left(0 ,1 ,\frac{1}{9} ,0, 0, 0, 0 ,0, 0, 0, 0\right)^{T}.\\ A= & {} \left( \begin{array}{llllllllllll} &{}9 &{}9 &{}2 &{}1 &{}0 &{}0 &{}0 &{}0 &{}0 &{}0 &{}0\\ &{}8 &{}1 &{}8 &{}0 &{}1 &{}0 &{}0 &{}0 &{}0 &{}0 &{}0\\ &{}1 &{}8 &{}8 &{}0 &{}1 &{}0 &{}0 &{}0 &{}0 &{}0 &{}0\\ &{}7 &{}1 &{}1 &{}0 &{}0 &{}0 &{}-1 &{}0 &{}0 &{}0 &{}0\\ &{}1 &{}7 &{}1 &{}0 &{}0 &{}0 &{}0 &{}-1 &{}0 &{}0 &{}0\\ &{}1 &{}1 &{}7 &{}0 &{}0 &{}0 &{}0 &{}0 &{}-1 &{}0 &{}0\\ &{}1 &{}0 &{}0 &{}0 &{}0 &{}0 &{}0 &{}0 &{}0 &{}1 &{}0\\ &{}0 &{}1 &{}0 &{}0 &{}0 &{}0 &{}0 &{}0 &{}0 &{}0 &{}1\\ \end{array}\right) , \\ \end{aligned}$$

### *Example 5*

(Refs. Wang et al. 2012; Thoai 1991).

$$\begin{aligned} \left\{ \begin{array}{ll} \min &{}(0.813396x_{1}+0.67440x_{2}+0.305038x_{3}+0.129742x_{4}+0.217796)\\ &{}\times (0.224508x_{1}+0.063458x2+0.932230x3+0.528736x4+0.091947)\\ {\mathrm {s.t.}} &{}0.488509x_{1} +0.063565x_{2} +0.945686x_{3}+ 0.210704x_{4} \le 3.562809, \\ &{} -0.324014x_{1} -0.501754x_{2} -0.719204x_{3} + 0.099562x_{4} \le -0.052215, \\ &{} 0.445225x_{1} -0.346896x_{2} + 0.637939x_{3} -0.257623x_{4} \le 0.427920, \\ &{} -0.202821x_{1} + 0.647361x_{2} + 0.920135x_{3} -0.983091x_{4} \le 0.840950, \\ &{} -0.886420x_{1} -0.802444x_{2} -0.305441x_{3} -0.180123x_{4} \le -1.353686, \\ &{} -0.515399x_{1} -0.424820x_{2} + 0.897498x_{3} + 0.187268x_{4} \le 2.137251, \\ &{} -0.591515x_{1} + 0.060581x_{2} -0.427365x_{3} + 0.579388x_{4} \le -0.290987, \\ &{} 0.423524x_{1} + 0.940496x_{2} -0.437944x_{3} -0.742941x_{4} \le 0.373620, \\ &{} x_{1} \ge 0, x_{2} \ge 0, x_{3} \ge 0, x_{4} \ge 0. \end{array} \right. \end{aligned}$$

### *Example 6*

(Refs. Chen and Jiao 2009).

$$\begin{aligned} \left\{ \begin{array}{ll} \min &{}(6x_1+x_2+1)(x_1+2x_2+1)+(-x_1+3)(x_1+x_2+1)\\ {\mathrm {s.t.}} &{} -2x_1+x_2\le 0,\\ &{} x_1+x_2\le 8,\\ &{} 0 \le x_1 \le 2.5,\\ &{} x_2 \ge 0. \end{array} \right. \end{aligned}$$

### *Example 7*

(Refs. Shen et al. 2008).

$$\begin{aligned} \left\{ \begin{array}{ll} \min &{}-4x^{2}_1-5x_2^{2}+x_1x_2+2x_1\\ {\mathrm {s.t.}} &{} x_1-x_2 \ge 0,\\ &{} \frac{1}{3}x_{1}^{2}-\frac{1}{3}x_{2}^{2}\le 1,\\ &{} \frac{1}{2}x_{1}x_{2}\le 1,\\ &{} 0 \le x_1 \le 3,\\ &{} x_2 \ge 0. \end{array} \right. \end{aligned}$$

### *Example 8*

(Refs. Shen and Jiao 2006; Jiao and Liu 2015).

$$\begin{aligned} \left\{ \begin{array}{ll} \min &{}x_1x_2-2x_{1}+x_2+1\\ {\mathrm {s.t.}} &{} 8x_2^{2}-6x_1-16x_2 \le -11,\\ &{} -x_{2}^2+3x_1+2x_2 \le 7,\\ &{} 1 \le x_1 \le 2.5,\\ &{} 1 \le x_2 \le 2.225. \end{array} \right. \end{aligned}$$

### *Example 9*

(Refs. Shen and Jiao 2006; Jiao and Liu 2015).

$$\begin{aligned} \left\{ \begin{array}{ll} \min &{}x_1 \\ {\mathrm {s.t.}} &{} \frac{1}{4}x_{1}+\frac{1}{2}x_{2}-\frac{1}{16}x^{2}_{1}-\frac{1}{16}^{2}x_{2}\le 1,\\ &{} \frac{1}{14}x^{2}_{1}+\frac{1}{14}x^{2}_{2}-\frac{3}{7}x_{1}-\frac{3}{7}x_{2}\le -1,\\ &{} 1 \le x_1 \le 5.5,\\ &{} 1 \le x_2 \le 5.5. \end{array} \right. \end{aligned}$$

### *Example 10*

(Refs. Jiao and Liu 2015).

$$\begin{aligned} \left\{ \begin{array}{ll} \min &{}x_1+(2x_1-3x_2+13)(x_1+x_2-1)\\ {\mathrm {s.t.}} &{} -x_1+2x_2\le 8,\\ &{} -x_2\le -3,\\ &{} x_1+2x_2\le 12,\\ &{} x_1-2x_2\le -5, \\ &{} x_1\ge 0,\\ &{} x_2\ge 0. \end{array} \right. \end{aligned}$$

### *Example 11*

Figure 1.

$$\begin{aligned} \left\{ \begin{array}{ll} \min &{}6x^{2}_1-x_2^{2}\\ {\mathrm {s.t.}} &{} -2x_1+x_2 \le 0,\\ &{} x_1+x_2 \le 8,\\ &{} \frac{1}{64}x_{1}^2-\frac{1}{64}x_{1}x_{2}\le 1,\\ &{} \frac{1}{4}x_{1}x_{2}-\frac{1}{8}x_{2}^2\le 1,\\ &{} -5 \le x_1 \le 5,\\ &{} -5 \le x_2 \le 5. \end{array} \right. \end{aligned}$$

### *Example 12*

$$\begin{aligned} \left\{ \begin{array}{ll} \min &{}f(x)=\sum \nolimits _{i=1}^p(a_{0i}^Tx+d_{0i})(c_{0i}^Tx+e_{0i})\\ {\mathrm {s.t.}} &{} \sum \nolimits _{i=1}^p(a_{1i}^Tx+b_{1i})(c_{1i}^Tx+d_{1i}) \le 0,\\ &{} \sum \nolimits _{i=1}^p(a_{2i}^Tx+b_{2i})(c_{2i}^Tx+d_{2i}) \ge 0,\\ &{} x \in D=\{x \in R^n \mid Ax \le b\}. \end{array} \right. \end{aligned}$$

where the real numbers $a_{ij},c_{ij},d_{ij}$ and $e_{ij}$ are randomly generated in the range $[-1,1],$ the real elements of *A* and *b* are randomly generated in the range [0, 1]. For this problem, we tested twenty different random instances and listed the computational results in Table 2, where the notations used in the head line have the following means: Iter:average numbers of iterations in the algorithm; Time: average CPU time in seconds; *m* and *n* denote the number of linear constraints and variables, respectively.

**Table 2:** Numerical results of Example 12

*p*	*m*	*n*	Iter	Time
5	10	10	8.8	5.0245
5	10	20	10.3	7.2315
5	10	30	28.0	45.1347
10	10	20	37.4	65.0159
10	20	40	49.2	75.1032

## Concluding remarks

In this study, a new global optimization algorithm is presented for solving generalized linear multiplicative programming problem with multiplicative constraints. This method has three main features. First, the relaxation problem performs well in approximation effect. Second, to obtain the lower and upper bounds of the optimal value, we only need to solve some linear programming problems. Finally, the problem we investigated is more general than those in many other literatures and results of numerical contrast experiments show that our method performs better than those methods.
